# Permian metabolic bone disease revealed by microCT: Paget’s disease-like pathology in vertebrae of an early amniote

**DOI:** 10.1371/journal.pone.0219662

**Published:** 2019-08-07

**Authors:** Yara Haridy, Florian Witzmann, Patrick Asbach, Robert R. Reisz

**Affiliations:** 1 Museum für Naturkunde, Leibniz-Institut für Evolutions- und Biodiversitätsforschung, Berlin, Germany; 2 Institut für Radiologie, Charité—Universitätsmedizin Berlin, Berlin, Germany; 3 International Center of Future Science, Dinosaur Evolution Research Centre, Jilin University, Changchun, China; 4 Department of Biology University of Toronto Mississauga, Mississauga, Ontario, Canada; Chinese Academy of Sciences, CHINA

## Abstract

Bone remodeling is an essential physiological process in growth and healing. In modern systems deviations from normal bone physiology in the form of pathologies aid in the understanding of normal bone metabolism. Here we use external morphology and X-ray microtomography to diagnose and describe a metabolic bone disease in an amniote from the early Permian. The specimen consists of two fused tail vertebrae of a small varanopid from early Permian (289 million years old) cave deposits near Richards Spur, Oklahoma, USA. Inspection of the outer morphology reveals that the fusion encompasses the vertebral centra, zygopophyses and haemal arches, with the fusion zones distinctly swollen on the left side of the specimen. With visualization of its internal structure by microCT, this specimen is diagnosed as a complex metabolic bone disease. The radiological imaging suggests a pathologically high bone turnover rate, as shown by abnormal bone formation in some areas and increased bone resorption in others. This supports that the varanopid suffered from a metabolic bone disease similar to Paget’s disease of bone as seen in humans today, which is linked to both genetic and viral factors. This finding extends the occurrence of Paget-like disease to the early Permian, and–provided a viral component was present–would also be by far the oldest evidence of viral infection in the fossil record.

## Introduction

The Dolese quarry at Richards Spur is a famous fossil locality that yields the most diverse terrestrial Palaeozoic tetrapod assemblage worldwide with more than 40 tetrapod taxa [1]. The locality is situated near Fort Sill, Oklahoma (USA) and consists of a series of fissure fills in the Ordovician Arbuckle limestone [1–6]. The fissure fill sediments are early Permian (Artinskaian,Wolfcampian) in age (~289 million years old) and consist of soft clays and mudstone. They contain articulated specimens or isolated bones of mostly small to medium sized terrestrial tetrapods. Amniotes comprise numerous reptiles (parareptiles, captorhinomorphs, and diapsids) and synapsids (caseids, varanopids, sphenacodontids), and non-amniotes are represented by stem-tetrapods (aïstopods), amphibians (dissorophoid temnospondyls), and stem-amniotes (seymouriamorphs, “microsaurs,” and diadectids) (2,6–9). In this study, we describe the isolated find of two pathologically fused caudal vertebrae of a varanopid from Richards Spur.

Varanopids range from the latest Carboniferous to the latest Middle Permian with a broad geographic distribution in Pangea. They were small to medium-sized (1.5 – 2m total body length) predators, superficially resembling extant varanids (monitor lizards) in their habits [6]. Varanopids are generally considered to be basal pelycosaur-grade synapsids [6–8], but a recent study has raised the possibility that they might represent basal diapsid reptiles [9]. Two varanopid taxa have been found at Richards Spur on the basis of fragmentary materials, the mycterosaurine *Mycterosaurus* [10] and the varanodontine *Varanops* cf. *V*. *brevirostris* [2,11]. The fossil material described here is part of a large recent collection of articulated and disarticulated specimens of a new undescribed third varanopid, one that appears to be closely related to *Mycterosaurus*.

In this study we present a description and diagnosis of two pathologically fused caudal vertebrae of an undescribed varanopid based on outer morphology and the internal microstructure as revealed by μCT. The specimen likely suffered from a metabolic bone disease that closely resembles Paget’s disease of bone, a metabolically active bone disease with high bone turnover rates, i.e. abnormal bone resorption by osteoclasts and enhanced abnormal bone formation by osteoblasts [12–14]. Because the etiology of Paget’s disease of bone is usually thought to have a viral component [15,16], the pathological varanopid vertebrae presented here might be the earliest indirect evidence of virus in the fossil record. As discussed below, an alternative diagnosis, fibrous dysplasia, cannot be ruled out, although is less likely. To our best knowledge, no other pathologies have so far been described in varanopids.

## Material

The material investigated here consists of two fused (MB.R.5931), and one undistorted vertebra (MB.R.5932) with minimal taphonomic wear as is typical for many fossils from the Richards Spur locality. The specimens are housed at the Museum für Naturkunde. The collective anteroposterior length of the fused vertebrae is 23 mm. Taking the midpoint of the ventral fusion zone between the centra as the boundary between the two elements, the anterior vertebra has a length of 11 mm and the posterior one of 12 mm. The vertebrae can be assigned to Varanopidae based on the proportionally elongate, slender vertebral bodies with a small perforating foramen in the mid-portion of the centrum and the double keelation on the ventral surface. In addition, the neural arches are slender and slightly concave, and the neural spines are delicate, and posterodorsally slanted. Both specimens are readily identifiable as caudal vertebrae because the zygapophyses are located close to the midline, and there are no parapophyses or transverse processes for ribs. Several normal caudal vertebrae were externally examined for comparison, and one was scanned for comparisons of the internal morphology.

## Methods

Measurements were taken using ImageJ, the trabecular thickness was measured at the anterior most slice on the anterior region of the centra. Nine random measurements were taken and then averaged to give a mean trabecular thickness for both the normal and the pathological vertebrae. The cortical bone thickness was measured in cross section of the midpoint of both the normal and the pathological vertebrae and again in the longitudinal sections of both the normal and pathological vertebrae (see supplementary information).

Specimens were CT scanned at the Museum für Naturkunde Berlin using a GE Phoenix nanotom, the resulting three-dimensional volumes were analyzed using the visualization software Volume Graphics Studio MAX 2.2.

## Results

### External morphology

**Fig 1:**
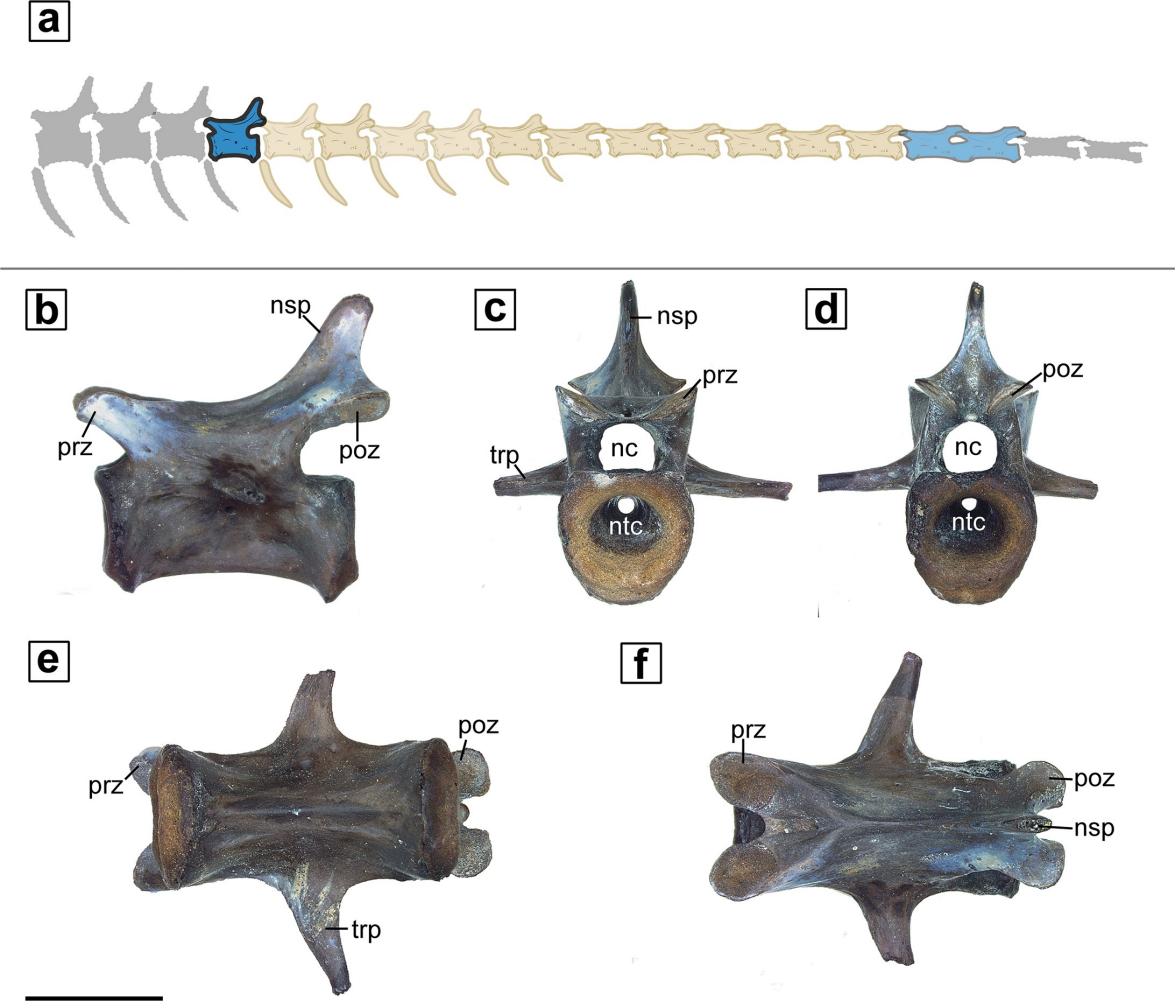
External anatomy of normal vertebrae (MB.R.5932). **a.** schematic of varanopid tail with the normal vertebrae highlighted; **b-f.** lateral, anterior, posterior, ventral and dorsal views respectively. **Abbreviations: nc,** neural canal; **nsp,** neural spine; **ntc,** notochordal canal; **prz,** prezygapophysis; **poz,** postzygapophysis; **trp,** transverse process. Scale bar = 5mm.

The external morphology of the vertebrae of varanopids has been described previously [2,6,11,17–19], so the description here is brief and focused on relevant features. Externally the normal vertebra is smooth and lacking any rugosity. It is identified as an anterior caudal by its low neural spine and the maintained presence of transverse processes (Fig 1), which are reduced and subsequently lost in more posterior caudal vertebrae. The more anterior caudal vertebrae maintain a single edged keel on their ventral surface, as opposed to the more posterior caudal vertebrae, which have two less pronounced keels. The articular surface of the centrum of the normal caudal specimen is predominantly comprised of ‘unfinished’ bone that would have likely been covered with cartilage. The centrum is characterized by the wide opening of the notochordal canal. The notochord was continuous throughout the element and was constricted mid-vertebra and then increased in diameter at the ends. The neural canal is rounded in cross section.

**Fig 2:**
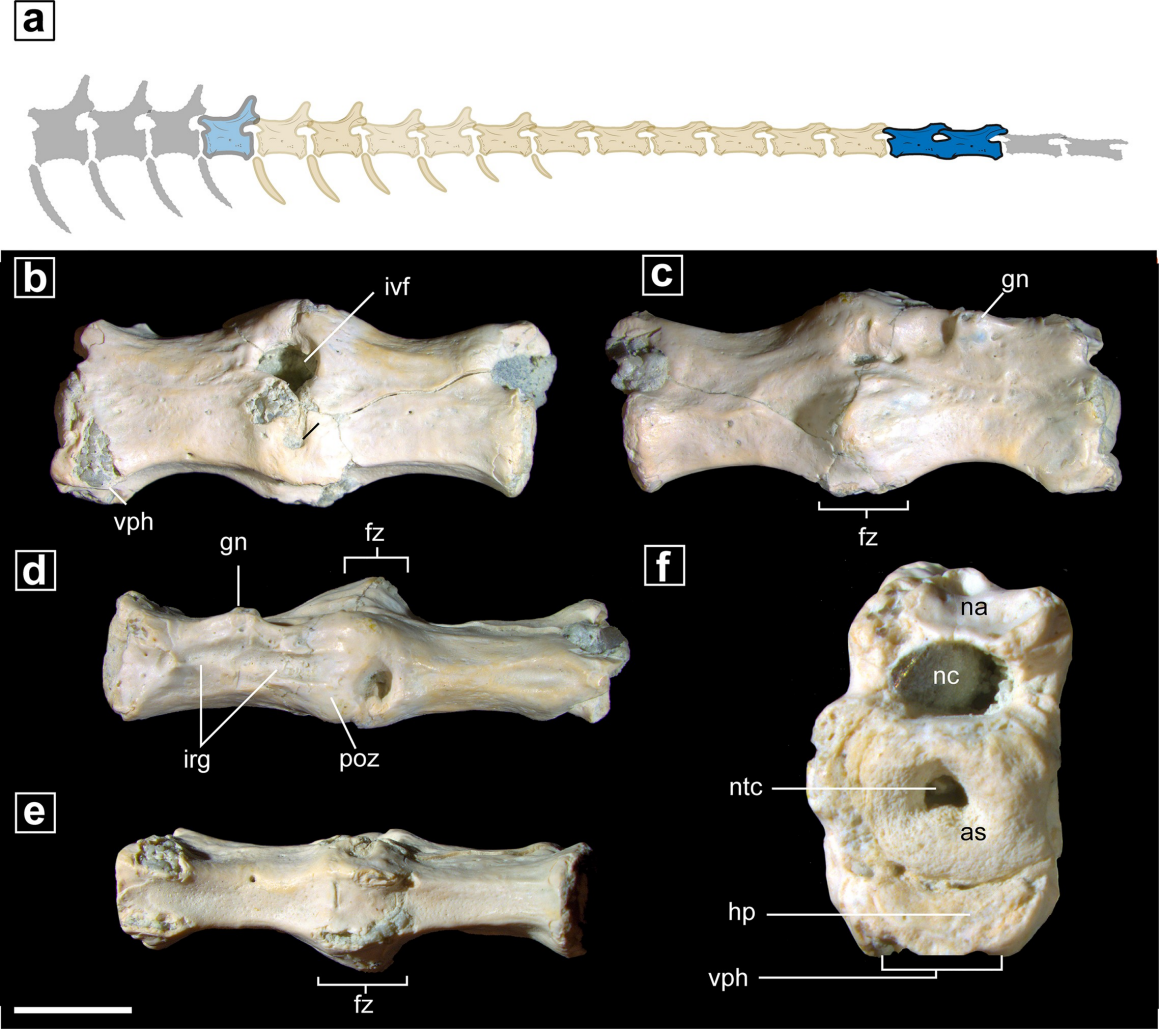
External anatomy of pathological varanopid vertebrae (MB.R.5931). **a.** schematic of varanopid tail with the normal vertebrae represented in blue, and the pathological fused vertebrae presented in orange.; **b-f.** right lateral, left lateral, dorsal, ventral, and anterior views respectively. **Abbreviations: as,** articular surface; **fz,** fusion zone; **gn,** growth nodule; **hp,** haemapophyses; **irg,** irregular groove; **ivf,** intervertebral foramen; **na,** neural arch; **nc,** neural canal; **ntc,** notochordal canal; **poz,** postzygapophysis fused; **vph,** ventral processes of haemapophyses. Scale bar = 5mm.

In the pathological specimen, the two vertebral centra are completely fused to one another without any superficial trace of an intervertebral suture (Fig 2). Additionally, the proximal part of a haemapophyses is fused to the posteroventral margin of the anterior centrum and to the anteroventral margin of the posterior centrum. Additionally, the proximal part of another haemapophysis is fused to the anteroventral margin of the anterior vertebra. The neural arches are completely fused to the centra such that there is no evidence of a neurocentral suture. The fusion zone between the centra protrudes outwardly and forms a large swelling of bone on the right side. In ventral view, the proximal part of the haemapophyses is visible and shows a left-right-asymmetry in the bases of their paired ventral processes; the base of the right process is larger than the left one and extends further anteriorly. In both haemapophyses, the ventral processes are broken off. On the right dorsolateral surface of the neural arch, a small osseous growth is present at about the mid-length of the anterior vertebra. The abnormal growth is penetrated by several small foramina on its dorsal side and has a distinct concavity on its posterior part. The pre- and postzygapophysis are fused, but the boundary between them is still discernable. The right postzygapophysis of the anterior vertebra is enlarged as compared to the left one, corresponding to the swelling of the centra in the fusion zone and the larger base of the haemapophyses on the right side. Accordingly, the right prezygapophysis is also larger than the left one in the posterior vertebra. Apart from the zygapophyses, the dorsal side of the neural arch of the posterior vertebra seems to be pathologically unaltered, whereas the dorsal side of the neural arch of the anterior vertebra bears many irregular depressions and crests. The bone surface is irregular on the left side of the centra, there are irregular grooves, depressions and crests on the right side, sometimes resembling the imprints of large vessels. The bone surface, however, is always smooth with some nutrient foramina penetrating the bone. The right intervertebral foramen for the spinal nerve root between the anterior and posterior vertebra is largely filled with bone; only two smaller openings of unequal size are present in the depression that represents the original foramen. From the larger of these openings, a shallow groove extends in a dorsal direction. In contrast, the intervertebral foramen on the right side is open but slightly constricted by bone growth on the anteroventral margin. A smaller opening is located ventrally on the lateral side of the swollen fusion zone of the centra, also showing a shallow groove running dorsally from the opening. The posterior edge of the posterior vertebra shows that at least the anterior margin of the intervertebral foramen between this and the posteriorly following vertebra was unaltered. The exposed (not fused) anterior and posterior articulation surfaces of the vertebral centra are concave (amphicoelous) and have a roughened, unfinished surface indicative of a cartilage cover in life. They are round in outline in anterior and posterior view, respectively, and have a centrally located, large notochordal canal. These joint surfaces were not pathologically altered. The opening of the neural canal is well preserved on the anterior face of the anterior vertebra. It is broad ovate in outline, measuring 2.5 mm in width and 1.5 mm in height, and is not constricted by pathological bone growth.

### Internal morphology

**Fig 3:**
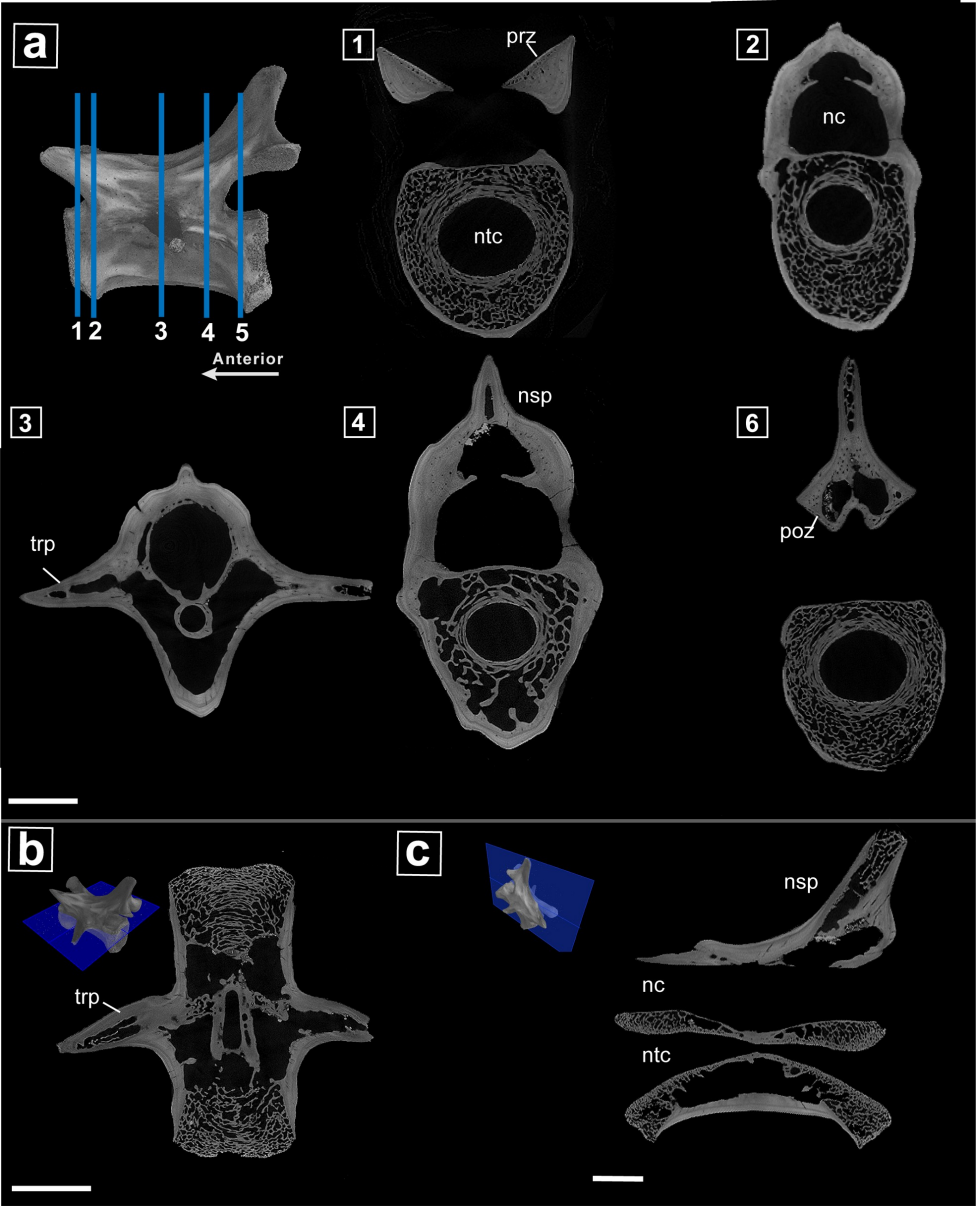
μCT internal anatomy of normal varanopid vertebra (MB.R.5932). **a**. (1–5) Serial cross sections showing the extent of the pathology throughout from most anterior to the posterior the two vertebrae; **b.** transverse section through the midline of the vertebra; **c.** sagittal section through the midline of the vertebra. **Abbreviations: nc,** neural canal; **nsp,** neural spine; **ntc,** notochordal canal; **prz,** prezygapophysis; **poz** postzygapophysis; **trp,** transverse process. Scale bar = 2mm.

The μCT scans of the normal vertebra (Fig 3) are provided here for comparison, and therefore their description is brief. The neural canal and notochordal canal are open with the notochord canal noticeably constricting towards the middle of the vertebrae (Fig 3A.3). The trabeculae are present only at the ends of the vertebra (Fig 3) and is almost completely absent in the midsection of the element (Fig 3A.3). The external cortex of the vertebra is made of layers of primary compact bone, which is likely lamellar bone that does not show any sign of resorption or remodeling. In both cross and longitudinal sections, lines of arrested growth (LAGs) can be seen in the cortex. The neural arch is taller in this specimen as it is more anterior in the caudal series, the trabeculae are restricted to the most dorsal portion of the neural spine and the rest of the bone appears compact.

**Fig 4:**
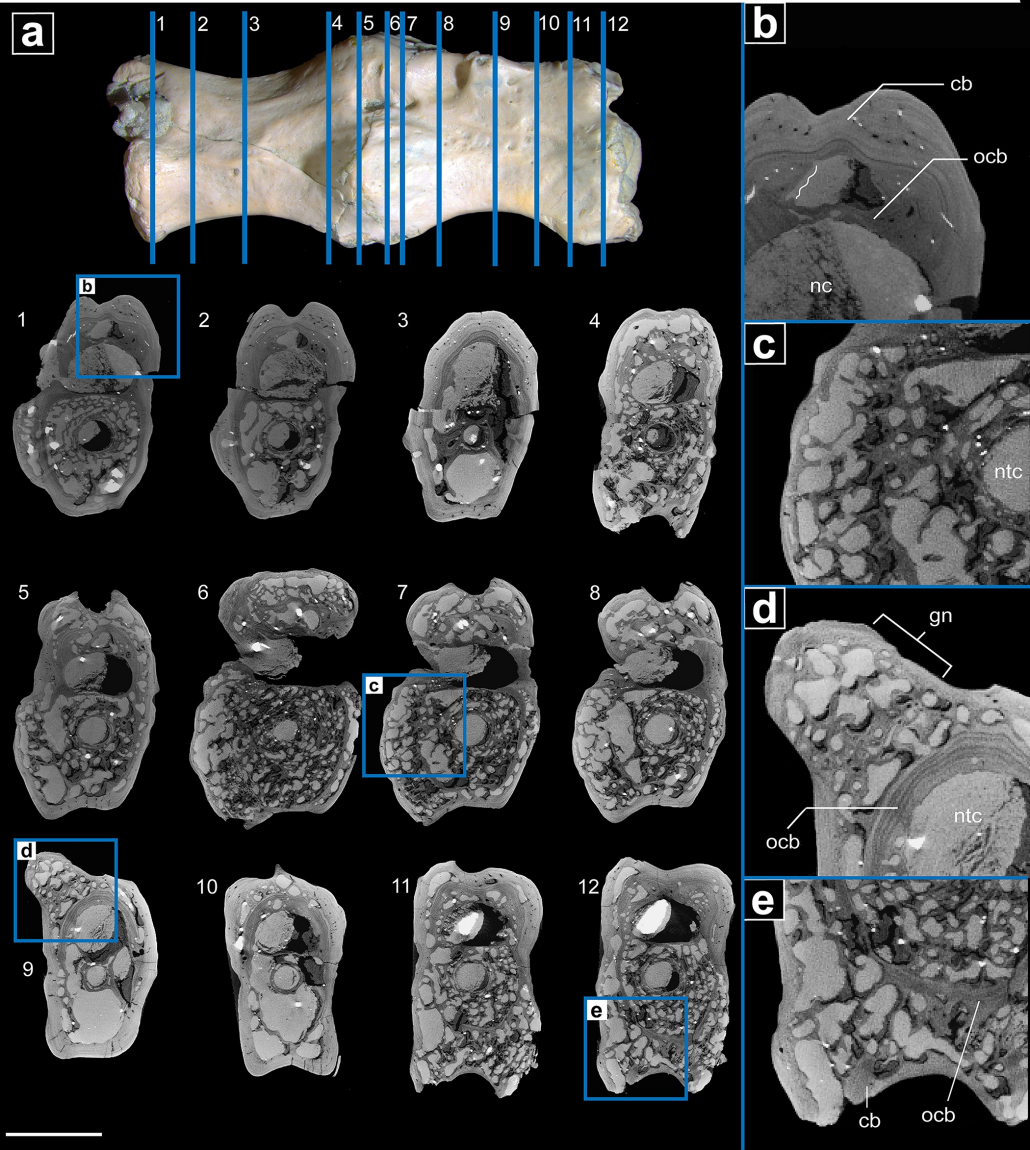
μCT internal anatomy of pathological varanopid vertebrae (MB.R.5931). **a.** Serial cross sections showing the extent of the pathology throughout from most anterior to the posterior the two vertebrae (1–12). **b.** closeup of cross section through neural arch showing the old cortical bone overlain by new less dense cortical bone; the wavy line shows the unevenness in the Howship’s lacunae. **c.** closeup of the outward-growth in the fusion zone showing lysis to the old cortical bone and thick erratic trabeculae overlain by a layer of cortical bone. **d.** closeup of the growth node on the second vertebrae’s neural arch, the growth overlays the old cortical bone and has thick trabeculae and large lytic spaces covered by cortical bone. **e.** closeup of the ventral portion of the second vertebrae showing the extent of resorption of old bone and deposition of new pathological bone. **Abbreviations: cb,** cortical bone; **ocb,** old cortical bone; **nc,** neural canal; **ntc,** notochordal canal. Scale bar = 2mm.

The μCT scans of the pathological fused vertebrae (Fig 4) show that the smooth exterior of the vertebrae did not indicate the extreme pathology that lay beneath the surface. The notochordal canal and the neural canal remain open and unobstructed in longitudinal and cross sections. However, the notochordal canal maintains a consistent diameter throughout both vertebrae, which is unlike the unaffected vertebrae in which the notochordal canal has a wider diameter towards the anterior and posterior ends. The cross-sectional outline of the neural canal is broad-ovate to reniform in the anterior and posterior regions of each vertebra. In the middle of the vertebra, it becomes nearly circular in cross section, as in the normal vertebra. In sagittal section, the neural canal expands at the boundary of the two vertebrae. The intervertebral space is almost absent and restricted to a very thin gap.

**Fig 5:**
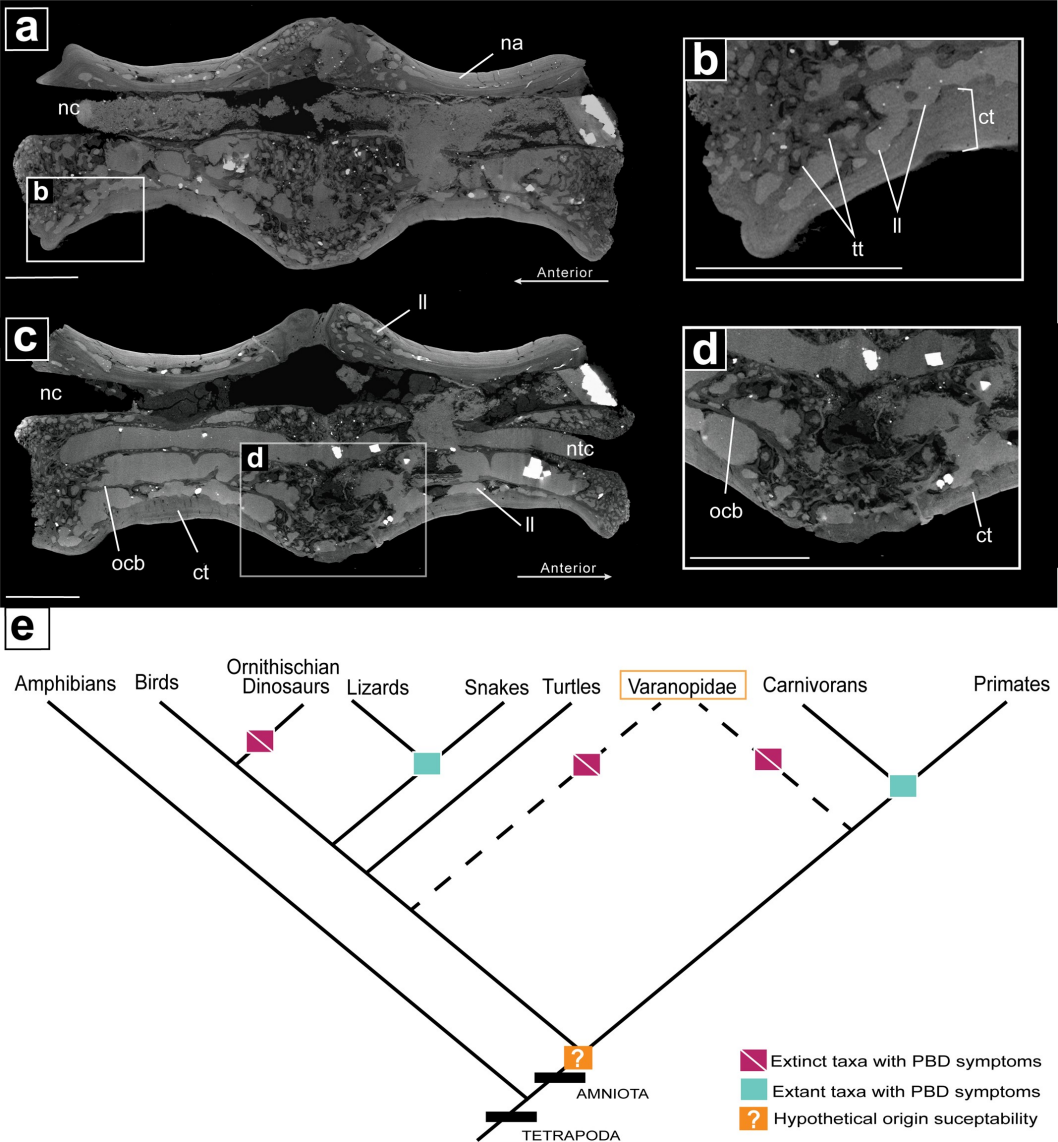
μCT internal anatomy of pathological varanopid vertebrae (MB.R.5931) and the phylogenetic prevalence on Paget like symptoms within amniotes. **a.** an off-centre sagittal section showing the internally altered pathological bone. **b.** a closeup of the anterior portion of vertebrae 1 showing thickened trabeculae, lytic lesions and cortical bone thickening. **c.** a centered sagittal section showing the notochord is still continuous through the pathological vertebrae, also showing the degree of alteration to the neural arch via lytic lesions; **d.** a closeup of the ventral region of the fusion zone, showing old cortical bone, large lytic lesions and cortical thickening; **e**. prevalence of Paget-like metabolic bone disease across tetrapods. **Abbreviations: ct,** cortical thickening; **ocb,** old cortical bone; **ll**, lytic lesion; **nc,** neural canal; **ntc,** notochordal canal; **tt,** thickened trabeculae. Scale bar = 2mm.

The μCT scans reveal in great detail the structure of the external cortical compact bone and of the inner trabecular bone of centra and neural arches. The original outer cortex of the vertebrae has been extensively altered through resorption and by addition of bone, and secondary formation of trabecular bone and cortical bone outside the original vertebral cortex has taken place. The cortical bone that surrounds the vertebrae is thickened, and there is a distinguishable difference between the old cortical bone that made up the original surface and the new cortical bone that covers the old cortex. The new cortical bone is denser and thus brighter in the μCT images (Figs 4 and 5), and the old cortical bone of the centrum and neural arch is covered in Howship’s lacunae, i.e. resorption bays, which are indicative of extensive osteoclastic activity. In some regions of the centrum, the old cortical bone has been so extensively resorbed that it is either a thin remnant covered in resorption bays or has been completely replaced by thickened trabeculae. The trabecular bone consists of thickened trabeculae in both the centra and the neural arch. Most trabeculae are present in about the anterior and posterior thirds of each vertebra, whereas the middle part is nearly devoid of trabeculae and consists of a large hollow space, as seen in the normal vertebra scans. The osseous bump mentioned above on the right side of the neural arch of the anterior vertebra consists of course trabecular bone with a thin external cortex, the trabecular bone is so pervasive that it continues into the neural arch, where the cortical bone has been resorbed and replaced with thickened trabecular bone (Figs 4 and 5).

In comparison with the normal vertebra, μCT shows that the trabeculae and external cortex of the fused vertebrae are thickened. The trabeculae of the fused vertebrae have a mean thickness of 0.077 mm, whereas the trabeculae of the normal vertebra have a mean thickness of 0.041 mm. The mean cortical thickness of the fused centra is 0.567 mm, whereas it is 0.313 mm in the centrum of the normal vertebra S1 Fig.

## Discussion

### Differential diagnosis

The differential diagnosis concerning the fused vertebrae describe above is as follows: (1) Infectious arthritis is rejected because not only the articular surfaces are involved, but also the entire vertebral bodies and neural arches [20]; (2) spondylitis tuberculosa is rejected because although there are lytic lesions in the varanopid vertebrae, spondylitis tuberculosa does not cause hyper-deposition of cortical bone or ankylosis[21]; (3) ankylosing spondylitis (spondyloarthropathy) is rejected because the intervertebral space is not maintained, and the entire vertebral body is involved in the pathology, not just the articulation points; there is extreme evidence of lysis in the varanopid, which is not a symptom of spondyloarthropathy [22]; (4) Scheuermann’s disease is rejected on the basis that there is no ventral narrowing of the vertebrae, no change in shape, and no Schmorl’s nodes [23]; (5) spondylitis ankylosans is very similar to the pathology described here due to the ankylosis of two or more vertebrae; however, there are no lytic regions associated with spondylitis ankylosans, and also secondary formation of trabecular bone and cortical bone outside the original vertebral cortex are not symptoms of this disease[24]; (6) vertebral tumor is rejected due to the lack of localization of the pathology, although there are two lytic growths on one side of the fused vertebrae, there are other symptoms (i.e. excessive cortical and trabecular bone growth outside the original cortex) that do not conform to a malignant or benign tumor [25]; (7) fracture callus causing the growth and subsequent fusion is rejected because there is overall cortical bone thickening, there is no disturbance of the old cortical bone except at the articulation point, yet the pathology encompasses at least two vertebrae [26]; (8) osteochondrosis intervertebralis is rejected because of an absence of spondylophytes and because the other vertebral body symptoms do not conform to a degenerative disease [27]; (9) chronic osteomyelitis is rejected on the basis of the reactive region that is not consistently lytic, the lamellar bone making up the outer cortex is laid down slowly and sequentially which is not congruent with infection. Osteomyelitis is also rejected on the basis of the external smooth appearance of the vertebrae, as osteomyelitis often causes a rugose and filigree appearance of bone [28].

However, fibrous dysplasia, which is one of the most important differential diagnoses for PDB, cannot be completely ruled out in the fused varanopid vertebrae. Fibrous dysplasia is a benign bone disorder in which mature, lamellar bone is not formed so that isolated trabeculae of immature (woven) bone can be found within dysplastic fibrous tissue or calcified cartilage [29–31]. Because the osteoblasts fail to differentiate and mature, fibrous dysplasia is considered a developmental disease. Radiologically, this classic form of fibrous dysplasia is well-defined and has a ground glass opacity as hallmark of the disease. This ground glass opacity refers to a homogeneous appearance on radiographs. Besides this classic form, a Pagetoid form of fibrous dysplasia exists which shows marked bone expansion or swelling similar to PDB. However, although we cannot definitely distinguish PDB and fibrous dysplasia here (because we do not have the typical picture frame vertebral body in the varanopid and due to the lack of soft tissue and biochemical markers), we consider PDB as more likely because most (70–80% [30,31]) cases of fibrous dysplasia are monostotic in humans whereas the varanopid pathology is polyostotic. Furthermore, the typical appearance of fibrous dysplasia has many thin delicate trabeculae seen in histological sections as well as radiographic imaging, giving the lesion the aforementioned pathognomonic ground glass appearance [29,31,32], and this is not the case here. Lastly, secondary degeneration and ankylosis of joints is not common in fibrous dysplasia [31].

### Taphonomy

In paleopathology, a differential diagnosis should include also so called “pseudopathologies”, meaning bone alterations that occurred after the death of the animal by taphonomic processes and may imitate true pathological alteration of the bone [33,34]. Such pseudopathologies are caused mainly by mechanical alteration during fossilization. For example, weathering and boring by insects may change the surface structure of the bones that may be mistaken for erosive diseases, and the weight of the overlying sediment may lead to cracks in the bone or even its plastic deformation, mimicking bone fracture in the living animal and osteomalacia (softening of bones by vitamin D deficiency), respectively. Furthermore, the imprints of plant roots on the bone surface may mimic the presence of abnormal blood vessels [30]. For the bone alterations in the varanopid vertebrae described here, taphonomic processes can be ruled out for the following reasons. (1) The micro-CT scans show that the swollen and fused parts of the bones are caused by deposition of new reactive bone and not by postmortem deformation of the bone or secondary mineral growth. (2) The observed bone resorption occurs inside the vertebrae with an intact outer surface, so the resorptive areas cannot be attributed to physical weathering or boring and gnawing by animals. (3) The pathologically altered bone surface is irregular but always smooth, ruling out postmortem erosion, and the foramina on the pathological bone surface do not show bite marks of insect mandibles at their rims so that postmortem boring can be excluded. The only taphonomic alterations that are visible are pyrite crystals which show up as bright white in the CT scans due to their high density. The aforementioned crystals will wither not alter the bone structure of completely break it apart, but do not account for the organic looking resorptive surfaces seen in the scans.

### Paget’s disease of bone

The radiological imaging of the two fused varanopid tail vertebrae described here shows thickening and enlargement of bone as well as pervasive large resorptive areas. This closely resembles the radiographic characteristics of Paget’s disease of bone (PDB) as described in humans^2^. PDB, also called osteitis deformans or osteodystrophia deformans, is a chronic, non-inflammatory, focal bone remodeling disease with pathologically increased bone turnover rates, i.e. enhanced resorption of bone is associated with accelerated, random new bone formation [12–14,35]. In PDB, the number and size of osteoclasts (bone-absorbing cells) is strongly increased with a higher number of nuclei per cell (up to 100), and their bone absorbing activity is enhanced, creating lytic areas and Howship’s lacunae in the affected bones[14,16].

Three major phases can be distinguished in PDB, the initial lytic phase, the following mixed phase, and the final osteoblastic phase [12]. The lytic phase is characterized by a reduction in bone density, caused by the aforementioned increase in osteoclast activity leaving lytic lesions and large spaces within trabecular bone. The mixed (or intermediate) phase is a combination of an increase in osteoblastic and osteoclastic activity where there is both excessive deposition and resorption occurring in the bone. Lastly, the osteoblastic phase, sometimes referred to as the sclerotic phase, is defined by an increase in osteoblastic activity resulting in thickening of the cortex and trabeculae.

In humans, PDB can affect different parts of the skeleton but is most commonly identified in the pelvis, followed by the vertebral column [12]. In human vertebrae, it is most often the mixed phase that is first recognized in radiographic imaging with thickening and rarefication of trabeculae and a thickening of the bone cortex, often leading to the characteristic ‘picture frame’ appearance of the vertebrae [12,13]. In the last, osteoblastic phase, bone density increases, and the cortex becomes sclerotic, leading to so called ‘ivory vertebrae’ appearance due to an increase in contrast on radiographs. Enlargement of the affected vertebra(e) is often, but not always, the case [12]. PDB can also affect facet joints in human vertebrae (Pagetic facet joint arthropathy), including loss of joint space and fusion of joints (ankylosis) by new bone formation [12,36]. In humans, PDB may also affect the intervertebral disc: resorption of the disc and its replacement by pagetic bone may lead to Pagetic Vertebral Ankyloses (PVA) [12,37,38].

The cause of PDB is still unknown although most research shows that there is a genetic predisposition for susceptibility to this disorder, especially connected with mutations of gene SQSTM1[13,35]. Epidemiological studies indicate declining prevalence rates of PDB in humans, and this suggests that also environmental triggers are interacting with the genetic predisposition [35]. Environmental factors that have been previously discussed include wood-fired heating, tobacco smoking, consumption of brains, rural life, and especially contact with farm or wildlife animals (Cundy & Bolland 2008 and references therein). Most recently, there is mounting evidence of strong links between PDB and various zoonotic viruses in humans, and has also been suggested in snakes [39] however the ultimate cause of PDB remains controversial [15,16]. Thus, Paget’s disease seems to be a combination of a genetic susceptibility and a change triggered by a virus that leads to this metabolic bone disease. Arguably the most interesting aspect of this pathology is its indication of normal bone metabolism being altered due to a possible link with a zoonotic virus.

The two fused varanopid vertebrae have a pagetic appearance in the following characteristics (compared with PDB in the human spine [12]): (1) Cortical bone of centra and neural arches thickened in comparison to the normal (see supplementary measurements); (2) lytic lesions present; (3) thickening or hypertrophy of bone trabeculae (see supplementary measurements); (4) pumice stone growth on the right lateral side of the anterior neural arch; (5) expansion of vertebral centrum, neural arch (zygapophyses) and haemapophyses especially on the right side; (6) no vertebral centrum expansion in direction of the synovial articular surfaces (corresponding to the mammalian endplates); and (7) increased bone density at the periphery. Also, the bony fusion between the two vertebrae (involving both centra and zygapophyses) and of two haemapophyses to the centra is consistent with a PDB-like appearance since pagetic facet joint arthropathy with bony fusion has been described in PDB [23]. However, the fusion of the centra in the varanopid cannot be referred to as Pagetic Vertebral Ankylosis (PVA) in the strict sense [23] because only mammals have vertebral centra connected by discovertebral junctions with a true intervertebral disc consisting of an outer anulus fibrosus, an inner nucleus pulposus, and the cartilaginous endplates. Reptiles and birds, in contrast, have synovial joints between the vertebral centra, structurally comparable to the zygapophyseal joints (see discussion in [40]). Only the synovial joint between the anterior and posterior centrum was likely affected by secondary degeneration, which ultimately caused the fusion of the centra. Although these comparisons are based on the morphological and radiological occurrence of PDB in humans, and reptilian physiology differs from that of mammals, [41] reported that pagetic vertebrae in a snake had a similar radiologic appearance to those described in humans. In this varanopid specimen, what is most likely preserved is a late mixed phase or the osteoblastic phase. This conclusion is based on the sclerotic-like thickening of the cortex which is associated with the blastic phase. However, it is impossible to tell if the extensive lytic lesions were still actively being resorbed, so a mixed phase cannot be ruled out.

How was the varanopid affected by the disease in life? In humans, PDB is often asymptomatic and is thus often only incidentally detected [14]. However, some symptoms may arise after several years, including back pain, compression fractures (because the rapidly formed pagetic bone is comparatively brittle), neural dysfunction (cord or nerve root compression in the spine), bone deformation, osteoarthritis, or deformation of otic capsule (may lead to the loss of hearing)[12–14,42]. In rare cases, pagetic bone may undergo neoplastic transformation to a malignant tumor, mainly osteosarcoma [13], which is rarely described in the fossil record[43]. The aforementioned symptoms have been recognized in humans (functionally bipedal animals), it shoulw be noted that little is known in how it may affect quadrupedal animals. Furthermore only the two fused vertebrae are preserved from the varanopid individual, so we cannot say if other parts of the skeleton were affected. Conceivably the disease could have caused some pain and restricted the flexibility of the tail to a certain degree. However, if only the distal part of tail was affected and no other skeletal regions such as the limb bones or the skull, the animal was probably not severely hampered by this pathology.

## Conclusions

Since soft tissues are not preserved in the fused varanopid vertebrae and therefore cannot be considered in the diagnosis, the pathology in the varanopid can best be referred to as “PDB-like condition”. The oldest PDB-like bone alteration has been reported in a Late Jurassic (150 million years old) vertebra of the dinosaur *Dysalotosaurus* [44]. If our interpretation for the pathologic varanopid is correct, then this would extend the record of this kind of disease far back to the early Permian (290 million years old), and–provided a viral component was present–would also be the earliest indirect evidence of virus in the fossil record. No other palaeontological evidence of PDB-like lesions exists in the fossil record. However, archaeological studies were able to detect PDB or PDB-like lesions in human remains dating back to the Neolithic period [45–47].

So far, PDB-like lesions have been reported among mammals in humans and other primates (macaques: [48]; lemurs and orangutans: [34]) and in dogs[49], and among reptiles in extant snakes[50,51], an extant lizard[52], and in a dinosaur[44]. This implies that the susceptibility for PDB-like disease must have evolved in early amniotes prior to the split in the synapsid (leading to mammals) and Diapsid (leading to reptiles, including birds) lineages. The find of a PDB-like lesion in a basal amniote, a varanopid, supports this assumption, irrespective of the question if varanopids represent basal synapsids or basal diapsids (Fig 5E).

Paget’s disease of bone in its essence is a bone metabolic disorder, an alteration of normal communication between bone building cells and bone destroying cells. However, while it is reasonable to assume that this normal communication has been present as far back as the first appearance of bone in the fossil record, its susceptibility to disruption is more difficult to ascertain. Disruption of normal physiology by a virus or other unknown factor can only be more thoroughly understood by studying pathological specimens, such as the one examined here which may suggest that similar gene mutations to those seen in Paget’s disease of bone were already possible in the Late Palaeozoic 290 million years ago.

## Supporting information

S1 Fig Figure indicating the plane and areas measured. Both the cortical thickness and trabecular thickness in both normal and pathological vertebrae (MB.R.5931 MB.R.5932) The measurements were taken using ImageJ.
(TIF)
