# Supplementary figures and images for: Permian metabolic bone disease revealed by microCT: Paget’s disease-like pathology in vertebrae of an early amniote
Source: PLoS One. 2019 Aug 7;14(8):e0219662. doi: 10.1371/journal.pone.0219662 (PMC6685605; doi:10.1371/journal.pone.0219662)

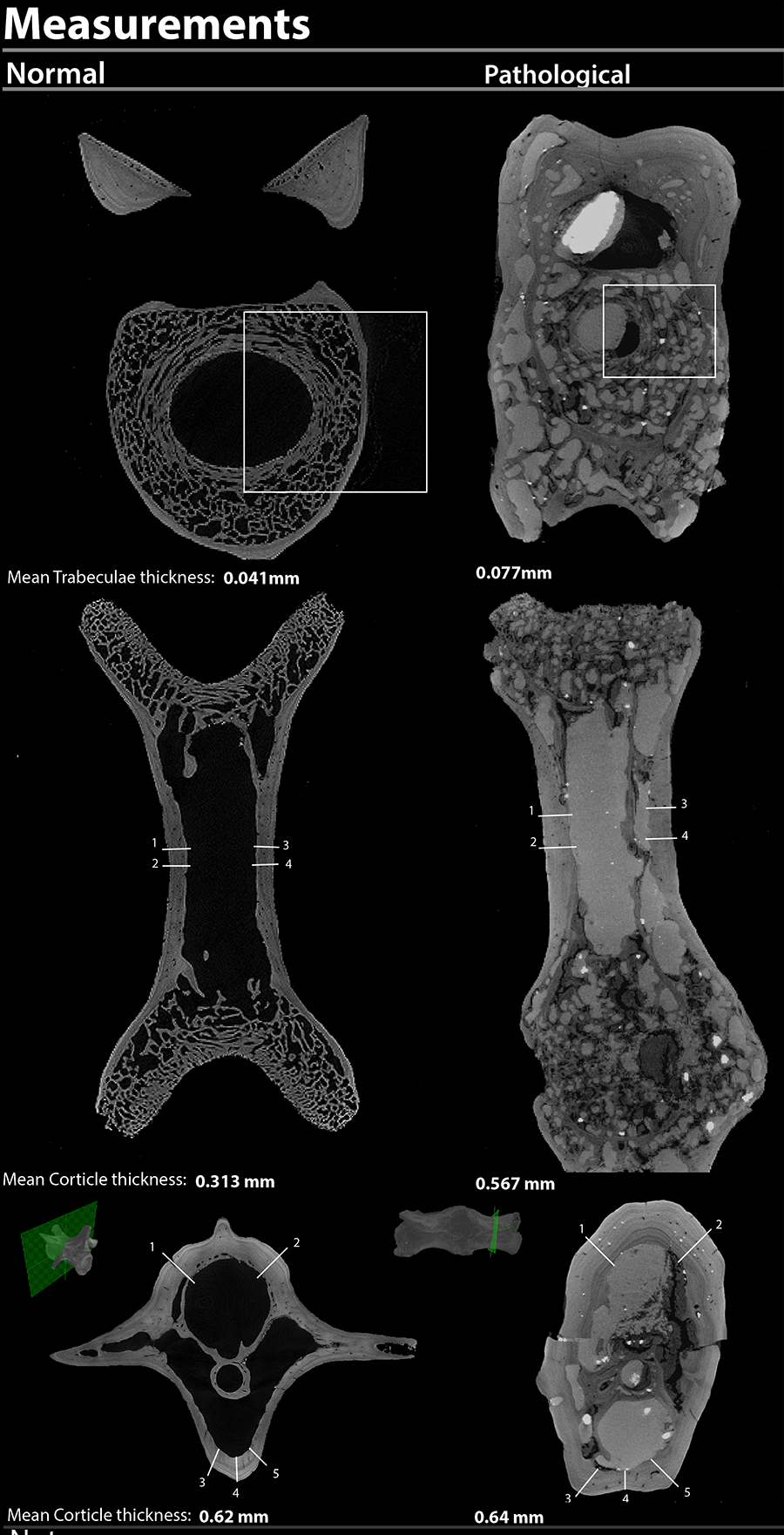

Supplement: S1 Fig — Both the cortical thickness and trabecular thickness in both normal and pathological vertebrae (MB.R.5931 MB.R.5932) The measurements were taken using ImageJ. (TIF) [file pone.0219662.s001.tif]
